# On finding bicliques in bipartite graphs: a novel algorithm and its application to the integration of diverse biological data types

**DOI:** 10.1186/1471-2105-15-110

**Published:** 2014-04-15

**Authors:** Yun Zhang, Charles A Phillips, Gary L Rogers, Erich J Baker, Elissa J Chesler, Michael A Langston

**Affiliations:** 1 Dupont Pioneer, Johnston, IA 50131, USA; 2 Department of Electrical Engineering and Computer Science, University of Tennessee, Knoxville, TN 37996, USA; 3 Department of Computer Science, Baylor University, Waco, TX 76798, USA; 4 The Jackson Laboratory, Bar Harbor, ME 04609, USA

**Keywords:** Biclique, Biclustering, Bioinformatics databases, Bipartite graphs, Graph algorithms

## Abstract

**Background:**

Integrating and analyzing heterogeneous genome-scale data is a huge algorithmic challenge for modern systems biology. Bipartite graphs can be useful for representing relationships across pairs of disparate data types, with the interpretation of these relationships accomplished through an enumeration of maximal bicliques. Most previously-known techniques are generally ill-suited to this foundational task, because they are relatively inefficient and without effective scaling. In this paper, a powerful new algorithm is described that produces all maximal bicliques in a bipartite graph. Unlike most previous approaches, the new method neither places undue restrictions on its input nor inflates the problem size. Efficiency is achieved through an innovative exploitation of bipartite graph structure, and through computational reductions that rapidly eliminate non-maximal candidates from the search space. An iterative selection of vertices for consideration based on non-decreasing common neighborhood sizes boosts efficiency and leads to more balanced recursion trees.

**Results:**

The new technique is implemented and compared to previously published approaches from graph theory and data mining. Formal time and space bounds are derived. Experiments are performed on both random graphs and graphs constructed from functional genomics data. It is shown that the new method substantially outperforms the best previous alternatives.

**Conclusions:**

The new method is streamlined, efficient, and particularly well-suited to the study of huge and diverse biological data. A robust implementation has been incorporated into GeneWeaver, an online tool for integrating and analyzing functional genomics experiments, available at http://geneweaver.org. The enormous increase in scalability it provides empowers users to study complex and previously unassailable gene-set associations between genes and their biological functions in a hierarchical fashion and on a genome-wide scale. This practical computational resource is adaptable to almost any applications environment in which bipartite graphs can be used to model relationships between pairs of heterogeneous entities.

## Background

Bicliques have a long history of applications. The enumeration of maximal bicliques can be traced at least as far back as the seminal work reported in [1]. There the problem was defined in terms of rectangles, binary relations and concept lattices. Subsequent progress on concept lattices was surveyed in [2,3]. Algorithms for their identification were applied to the analysis of gene co-expression data in [4,5].

A variety of biological challenges can be addressed by finding maximal bicliques in bipartite graphs. Representative applications include biclustering microarray data [6-8], optimizing phylogenetic tree reconstruction [9], identifying common gene-set associations [10], integrating diverse functional genomics data [11], analyzing proteome-transcriptome relationships [12], and discovering patterns in epidemiological research [13]. Statistical approaches have been applied to some of these problems, but in many cases a discrete approach is beneficial or required because of the structure and diversity of the data under study.

Let us describe a few specific examples. Bicliques have been used in the analysis of gene expression data to represent subsets of genes and subsets of conditions, each pair with a high similarity score [6]. Graph theoretical approaches have been proposed in this setting to find bicliques in the resultant bipartite graphs that model genes and conditions with vertices, and co-expression levels with edge weights [7,8,14]. Bicliques have been used in phylogenetics to improve the accuracy of tree reconstruction [9]. Such a tree denotes evolutionary relationships among species thought to have a common ancestor. Data with no fewer than *k* genes sampled from no fewer than *m* species are extracted from sequence databases. This operation is equivalent to finding maximal bicliques with partition sizes at least *k* and *m*. Bicliques have been used in epidemiological research to identify sets of individuals who share common sets of features. Bipartite graphs can help capture relationships between organisms and a wide range of factors. Maximal bicliques are particularly useful in case-control studies involving categorical features such as genotypes and exposures [13].

Our work has been largely motivated by the computational demands of systems like GeneWeaver [11], a web-based software platform for the integration of functional genomics data. GeneWeaver includes a database containing lists of genes from diverse sources, along with descriptive metadata associated with these lists. Through gene homology, the lists can be combined across species such that genes on the lists are translated to a common reference. This enables the construction of a bipartite graph, with vertex partitions representing individual genes versus the gene lists. A suite of tools built on the enumeration of maximal bicliques and other bipartite analyses allows the user to identify groups of genes that are associated with related biological functions, all without any prior knowledge or assumption about such group associations. Efficiency and scalability are paramount, because real-time maximal biclique enumeration is required for web-based user-driven analyses, as well as for effective computations over the entire data repository.

### Problem

In each of the aforementioned applications involving an integration of multiple sets of genome-scale data, bipartite graphs can be used to represent relationships across pairs of heterogeneous data types. An interpretation of such a relationship is accomplished through an enumeration of maximal bicliques. Let us be precise about what this means. A bipartite graph is one whose vertices can be partitioned into a pair of non-empty, disjoint partitions such that no two vertices within the same partition are connected by an edge. Let *G* denote a bipartite graph, let *U* and *V* denote its two vertex partitions, and let *E* denote its edge set. A biclique in such a graph is a complete bipartite subgraph, that is, a bipartite subgraph containing all permissible edges. The notion is formalized as follows:

#### 

**Definition 1.** *Let G *= (*U* ∪ *V,E) denote a bipartite graph. A biclique C* = (*U*^′^,*V*^′^*) is a subgraph of G induced by a pair of two disjoint subsets U*^′^ ⊆ *U,V*^′^ ⊆ *V,* such that ∀ *u* ∈ *U*^′^*,v* ∈ *V*^′^*, (u,v*) ∈ *E.*

A *maximum biclique* is a largest biclique in a graph. Unlike the well-known maximum clique problem, there are two distinct variants of the maximum biclique problem: the *vertex maximum biclique* problem and the *edge maximum biclique* problem. The former asks that we find a biclique with the largest number of vertices, and can be solved in polynomial time [15]. The latter asks that we find a biclique with the largest number of edges, and is -complete [16]. In biological applications, the edge maximum biclique is often desirable because it models more balanced connectivity between the two vertex classes. For example, an edge maximum biclique may group together numerous related biological processes and a modest set of their common genes, whereas a vertex maximum biclique may instead group together only a tiny set of related biological processes with great numbers of common genes.

**Figure 1:**
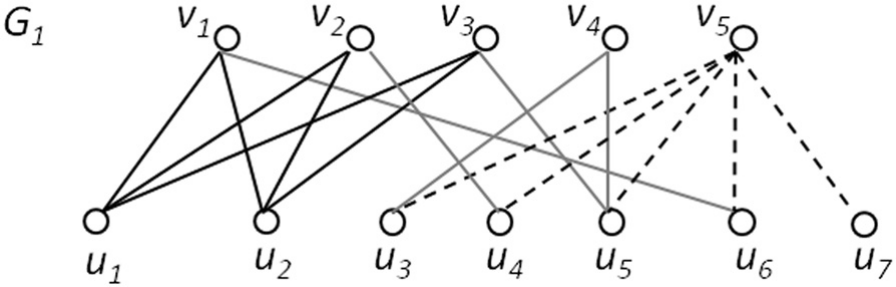
**Maximum and maximal bicliques.** A bipartite graph *G*_1_ has an edge maximum biclique *B*_1_({*u*_1_,*u*_2_},{*v*_1_,*v*_2_,*v*_3_}) with 5 vertices and 6 edges, and a vertex maximum biclique *B*_2_({*u*_3_,*u*_4_,*u*_5_,*u*_6_,*u*_7_},{*v*_5_}) with 6 vertices and 5 edges. Both *B*_1_ and *B*_2_ are maximal.

A *maximal biclique* is one not contained in any larger biclique. Examples of maximum and maximal bicliques are shown in Figure 1. The enumeration version of our problem is to find all maximal bicliques in a bipartite graph. In so doing, it turns out that we actually generate both edge maximum and vertex maximum bicliques. Thus, we are chiefly concerned with this enumeration problem, formalized as follows: 

**Input :** A bipartite graph *G* = (*U* ∪ *V*,*E*).

**Output:** All maximal bicliques, or subsets *U*^′^ of *U* and *V*^′^ of *V*, for which the induced subgraph *G*[*U*^′^ ∪ *V*^′^] is complete, and there are no subsets *U*^′′^ ⊇ *U*^′^ and $V^{\prime \prime }⊋V^{\prime }$, or $U^{\prime \prime }⊋U^{\prime }$ and *V*^′′^⊇*V*^′^, such that *G*[*U*^′′^∪*V*^′′^] is also complete.

As observed in [17], the maximal biclique enumeration problem cannot be solved in polynomial time since the number of maximal bicliques may be exponential in the graph size. Nevertheless, there remains a demand for efficiency, because we often need exact solutions to large-scale instances in real time. The Maximal Biclique Enumeration Algorithm (MBEA) that we will define here finds all maximal bicliques. It exploits structure inherent in bipartite graphs. It employs a branch-and-bound technique to prune non-maximal candidates from the search tree. Its pruning is accelerated by directly removing dominated vertices from the candidate set. Our experimental results demonstrate that the resultant reduction in search space enables MBEA to scale to the tens of thousands of nodes currently encountered in analyzing large biological data sets. In addition, we created an improved version, iMBEA, that selects candidate vertices in the order of common neighborhood size and that uses an enhanced version of branch pruning.

### Related work

With widespread applications such as those just discussed, one would expect a plethora of algorithms targeting maximal bicliques on bipartite graphs. Most algorithms that achieve this purpose, however, are either not tailored for bipartite graphs or not designed specifically for maximal biclique enumerations. Most existing graph algorithms for solving this problem fall into two main categories: (i) those designed for bipartite graphs but that either place undue restrictions on the input or require reduction to other problems, and (ii) those designed for general graphs and are thus unable to take advantage of bipartite graph structure. Table 1 lists these algorithms, their inputs and outputs (with restrictions, if any), and the methods they use.

**Table 1:** Previously presented graph algorithms for maximal biclique enumeration

**Algorithms**	**Inputs**	**Outputs**	**Methods**
Sanderson et al. [9]	Bipartite graph	Maximal bicliques of bounded minimum size	Exhaustive search by iterative biclique building
Mushlin et al. [13]	Bipartite graph	Maximal bicliques of bounded sizes and figure-of-merit values	Exhaustive search with a priority queue
Zaki et al. [21]	Bipartite graph	Maximal bicliques (one partition)	Frequent closed itemset mining in transaction databases
Uno et al. [25] (LCM/LCM2)	Bipartite graph	Maximal bicliques (one partition)	Frequent closed itemset mining
Li et al. [26] (LCM-MBC)	Bipartite graph	Maximal bicliques	Frequent closed itemset mining
Makino & Uno [18]	Bipartite graph	Maximal bicliques	Maximal clique finding in general graphs
Tomita et al. [30]	General graph	Maximal cliques	Maximal clique finding in general graphs
Eppstein [17]	General graph of bounded arboricity	Maximal bicliques	Exhaustive search
Alexe et al. [27] (MICA)	General graph	Maximal bicliques	A consensus algorithm
Liu et al. [28] (MineMBC)	General graph	Maximal bicliques of bounded minimum size	A divide-and-conquer approach

#### Algorithms for bipartite graphs

Existing algorithms for finding maximal bicliques in bipartite graphs are further divided into the following three approaches: exhaustive search with restrictions on outputs, reduction to the clique enumeration problem on general graphs, and reduction to the frequent itemset mining problem in transaction databases.

The most intuitive approach entails exhaustively building all subsets of one vertex partition, finding their intersections in the other partition, and checking each for maximality. Algorithms based on exhaustive search must generally place one or more restrictions on the problem to reduce its enormous search space. Moreover, exhaustive search requires storing generated bicliques to determine their maximality. An iterative algorithm is presented in [9] to build subsets progressively, from pairs of vertices to collections of larger and larger sizes. It limits the sizes of both biclique vertex partitions, yet still requires enormous amounts memory to store the lists used to generate subgraphs and decide maximality. The algorithm described in [13] builds bicliques based on set expansion and extension operations. It employs a hash table that determines maximality to avoid pairwise biclique comparisons, and a queue to maintain bicliques prioritized by figure-of-merit values (e.g., *p*-values). Users can specify constraints on the figure-of-merit values to filter out bicliques of insufficient interest.

**Figure 2:**
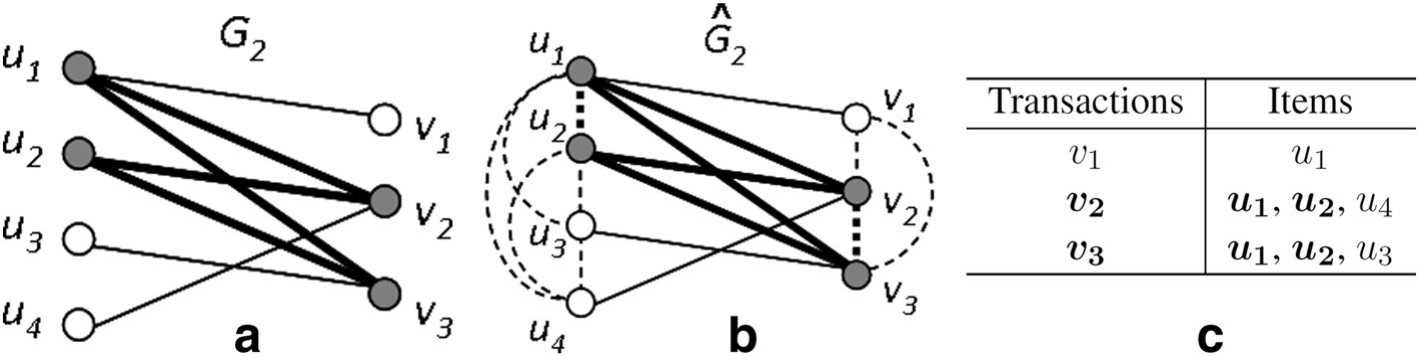
**Bicliques, cliques, and frequent closed itemsets.** The relationship between bicliques in a bipartite graph, cliques in a general graph, and frequent closed itemsets in a transaction database is exemplified. In **(a)**, the bipartite graph *G*_2_ has a maximal biclique *B* = ({*u*_1_,*u*_2_},{*v*_2_,*v*_3_}). In **(b)**, *G*_2_ has been transformed into $\hat{G_{2}}$ by adding edges (dashed lines) between every pair of nodes in the same partition. The vertices of *B* now form a maximal clique in $\hat{G_{2}}$. In **(c)**, a transaction database is built from *G*_2_ by treating *V* as the transaction set and *U* as the item set. *B* can now be viewed as a frequent closed itemset in this database.

The second approach relies on graph inflation. As observed in [18], the enumeration of maximal bicliques in a bipartite graph can be transformed into the enumeration of maximal cliques in a general graph by adding all possible edges between vertices within the same partition, thereby transforming each of the two disjoint vertex sets into a clique. This approach is neither practical nor scalable, however, due to the enormous number of edges that may be needed and the concomitant increase in problem difficulty that is incurred. Given a bipartite graph *G* = (*U* ∪ *V*,*E*) where |*U*| = *m*, |*V*| = *n*, |*E*| = *e*, the number of edges needed to transform *G* to a corresponding graph $\overset{ˆ}{G}$ is n2+m2. Thus, this method transforms the problem of finding maximal bicliques in a bipartite graph with edge density $d(G)=\frac{e}{m\times n}$ to the problem of finding maximal cliques in a graph $\overset{ˆ}{G}$ with density d(Gˆ)=e+n2+m2m+n2. Note that $\overset{ˆ}{G}$ might be dense even if *G* is sparse. When *G* has two vertex sets of equal size and no edges (i.e. |*U*| = |*V*| = *n*, |*E*| = 0), $\overset{ˆ}{G}$ has a density $\frac{n^{2}-n}{2n^{2}-n}≃50\%$. Figure 2(a,b) illustrates the correspondence between these two problems.

A third approach comes from the field of data mining. It was observed in [19] that a transactional database can be represented by a bipartite graph, with a one-to-one correspondence between frequent closed itemsets and maximal bicliques. A subset of items is defined as a *frequent itemset* if it occurs in at least one transaction. On one hand, a frequent itemset and the set of transactions containing the frequent itemset form a biclique. On the other hand, the adjacency lists of a bipartite graph can be viewed as a transaction database by treating each vertex in one set as an item and each vertex in the other set as a transaction that contains a subset of items. A biclique can thus be mapped to a frequent itemset. A maximal biclique corresponds to a frequent closed itemset, where a frequent itemset *I* is said to be *closed* if the set of transactions containing *I* do not contain a superset of *I*. The *support* of a frequent itemset is the number of transactions in which the set occurs. Enumerating all maximal bicliques is equivalent to enumerating all frequent closed itemsets with support at least 1. Figure 2(a,c) shows a mapping between these two problems. A correspondence between maximal bicliques of a general graph and frequent closed itemsets has been shown [20], leading to the suggestion that FPclose and similar frequent itemset mining methods [21-25] may be helpful in enumerating maximal bicliques. Implementations of this approach require a post-processing step to obtain the transaction set for each frequent closed itemset, as described in [26]. This is because the published methods output only the frequent itemsets (which correspond to half bicliques). Although this post-processing step is straightforward enough, it can be prohibitively time-consuming when the number of maximal bicliques is large. Moreover, known methods take the support level as an input parameter, and find only frequent closed itemsets above the given support. (In general, the lower the support, the longer the algorithms take. A support of 1 is the most difficult, since at this level all frequent closed itemsets must be found.)

#### Algorithms for general graphs

Maximal bicliques can also be found with algorithms designed for general graphs. Such algorithms of course lack any efficiency gains that might be accrued from utilizing bipartite graph structure. The maximal biclique enumeration problem was studied from a theoretical viewpoint in [17], where the focus was on graphs of bounded arboricity. It was proved that all maximal bicliques in a graph of order *n* and arboricity *a* can be enumerated in *O*(*a*^3^2^2*a*^*n*) time. This approach is not practical for large graphs, however, because it is unrealistic to expect that arboricity would be limited in practice [19]. A suite of consensus algorithms was presented in [27] for finding complete bipartite (but not necessarily induced) subgraphs. Unfortunately, these algorithms need to keep all maximal bicliques in memory. The Modular Input Consensus Algorithm (MICA), the most efficient among them, has space complexity O(B) and time complexity $O(n^{3}B)$, where  denotes the number of maximal bicliques. An algorithm (MineLMBC) based on divide-and-conquer was proposed in [28] to mine large maximal bicliques from general graphs by putting size constraints on both vertex sets to iteratively prune the search space. The algorithm reduces the space complexity to *O*(*n*^2^) and the time complexity to $O(n^{2}B)$. The algorithm on dense graphs from 2nd DIMACS Challenge benchmarks outperforms MICA when minimum biclique sizes are constrained by certain thresholds.

To solve the biclique enumeration problem, restrictions on either inputs or outputs have been proposed to reduce the search space. These include bounding the maximum input degree [7], bounding an input’s arboricity [17], and bounding the minimum biclique size [9,28] or figure-of-merit [13]. Naturally, no algorithm relying on these restrictions can solve arbitrary bipartite instances.

## Methods

The algorithm we shall now describe achieves efficiency without I/O or other restrictions. Performance testing on both synthetic and biological graphs demonstrate that it is markedly superior to MICA [27], the best known algorithm for finding bicliques on general graphs, and to LCM-MBC [26], a state-of-the-art frequent itemset algorithm that improves upon and adds a post-processing step to LCM [25]. The Maximal Biclique Enumeration Algorithm (MBEA) combines backtracking with branch-and-bound techniques to prune away regions of the search tree that cannot lead to maximal bicliques. MBEA is inspired by the classic maximal clique-finding method of [29], which was refined and shown to have optimal time complexity in [30]. The search space for MBEA is limited to disjoint vertex sets because, in a biclique, vertices in one set determine those in the other.

### Algorithmic basics

Let *G* = (*U* ∪ *V*,*E*) be a bipartite graph and assume, without loss of generality, that |*U*| ≥ |*V*|. MBEA operates on the (potentially smaller) set *V*, utilizing the following four dynamically changing sets of vertices: (i) *R*, a subset of *V*, (ii) *L*, a subset of *U* containing all the common neighbors of *R*, (iii) *P*, a subset of *V* containing candidate vertices that may be added to *R*, and (iv) *Q*, a subset of *V* containing former candidates, that is, vertices that were previously considered for *R*. The sets *R*, *L*, *P* and *Q* are employed in a depth-first traversal of a recursion tree to form maximal bicliques. *R* and *L* are used to form such a biclique, where *R* determines *L*. *P* is used for biclique expansion. *Q* is used to determine maximality. *P*, *Q* and *R* are required to satisfy the following two conditions:

• (*P* ∩ *Q*) ∪ (*P* ∩ *R*) ∪ (*Q* ∩ *R*) = ∅. That is, *P*,*Q*,*R* are pairwise disjoint.

• *P* ∪ *Q* = {*v*|*v* ∈ *V*∖*R*, ∃ *u* ∈ *L*, (*u*,*v*) ∈ *E*}. That is, *P* and *Q* contain every vertex in *V* but not *R* that is adjacent to at least one vertex in *L*.

#### 

**Observation 1.** *The subgraph induced by (L,R) is a biclique.*

For simplicity, and since *G* is bipartite, we henceforth drop the reference to induced subgraph, and simply say that (*L*,*R*) is a biclique. Note that (*L*,*R*) is maximal iff there is no vertex in *U*∖*L* that is adjacent to all vertices in *R* and no vertex in *V*∖*R* that is adjacent to all vertices in *L*. Because *L* is defined by *R*, only the maximality of *R* need be considered.

#### 

**Observation 2.** *(L,R) is maximal iff no vertex in V*∖*R is adjacent to every vertex in L.*

If *P* contains a vertex that is adjacent to all vertices in *L*, then (*L*,*R*) is not maximal. Thus that vertex may as well be moved from *P* to *R*. This process can be iterated until no more vertices can be so moved. On the other hand, if none of the elements of *V*∖*R* is a common neighbor of all vertices in *L*, then (*L*,*R*) is maximal because *L* and *R* are the largest set of common neighbors of each other.

#### 

**Observation 3.** *Let S denote* {*v* | *v* ∈ *P* & (*u*,*v*) ∈ *E* ∀ *u* ∈ *L*}. *Then* (*L*,*R* ∪ *S*) *is a maximal biclique.*

If *Q* contains a vertex that is adjacent to all vertices in *L*, then not only (*L*,*R*) is not maximal, but also there can be no *S* as defined above for which (*L*,*R* ∪ *S*) is maximal. We can actually say slightly more than this, as follows.

#### 

**Observation 4.** *Let T denote* {*v* | *v* ∈ *Q* & (*u*,*v*) ∈ *E* ∀ *u* ∈ *L*}, *L*^′^ *denote any subset of L, and S*^′^ *denote any subset of P. Unless T is empty,* (*L*^′^,*R* ∪ *S*^′^) *is not a maximal biclique.*

**Figure 3:**
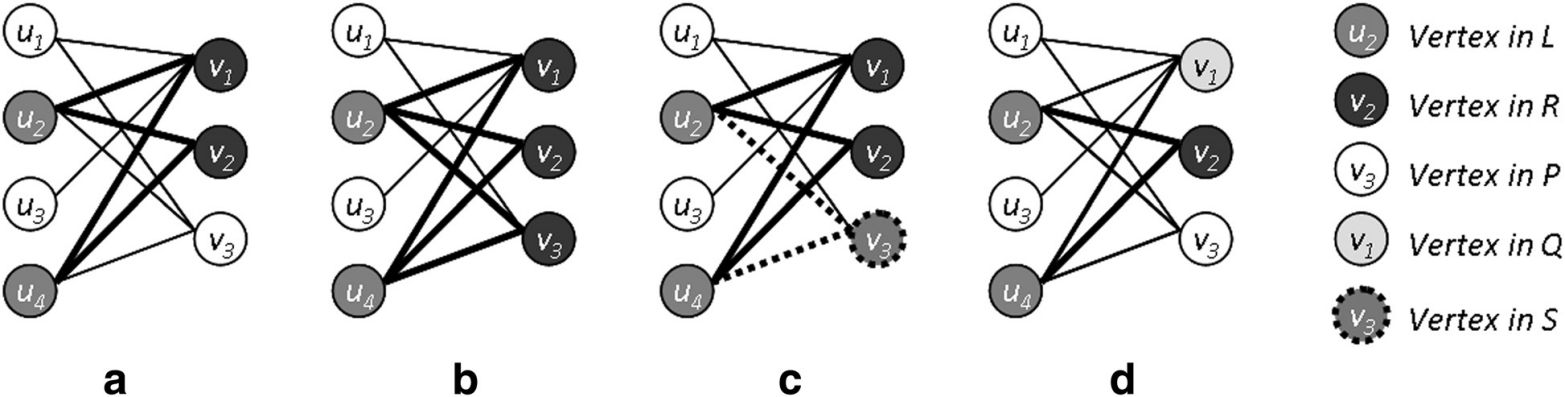
**Algorithmic observations.** Examples are shown to illustrate **(a)** Observation 1, **(b)** Observation 2, **(c)** Observation 3, and **(d)** Observation 4.

Observation 4 is used to prune unproductive subtrees in a branch-and-bound style exploration of the maximal biclique search space. As Observation 2 shows, if *Q* contains a vertex *v* adjacent to all vertices in *L*, it means that biclique (*L*,*R*) is not maximal. We further observe that none of the bicliques extended from *R* contains *v*, since *R* does not contain *v*. However, *v* is adjacent to all vertices in any subset of *L*. Thus, no bicliques extended from such a node is maximal and its subtrees can be pruned away. The utility of these observations is explicated in Figure 3.

### Algorithmic description

**Figure 4:**
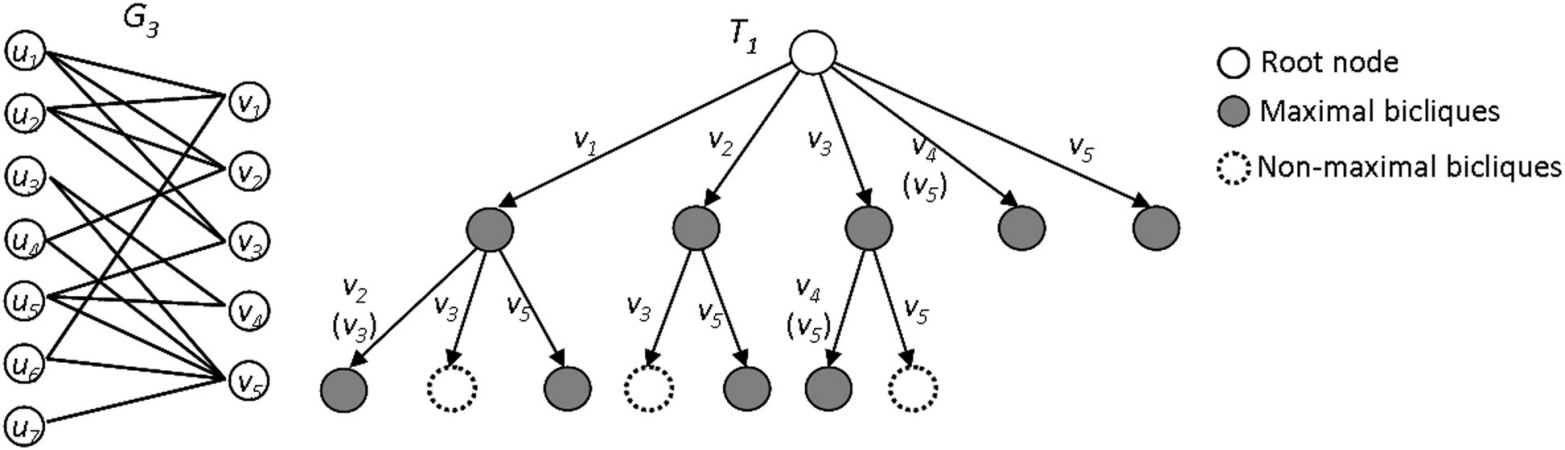
**The MBEA recursion tree.** The MBEA recursion tree *T*_1_ for a bipartite graph *G*_3_. The vertices in parentheses on the paths are those added by Observation 3.

To aid discussion, we refer the reader to pidgin pseudocode for Algorithm: MBEA. (For the time being we shall ignore starred lines that describe subsequent improvements.) Overall, a depth-first search tree traversal is performed recursively using the core function biclique_find(). Initially, all vertices are biclique candidates (*P* = *V*,*L* = *U*), while the biclique and former candidate sets are empty (*R* = *Q* = ∅). As the computation proceeds, *R* grows but *L* and *P* shrink. At each node of the search tree, biclique_find takes as input a 4-tuple 〈*L*,*R*,*P*,*Q*〉 and selects a candidate *x* from *P*. An extension step augments *R* with *x* to form *R*^′^, and forms *L*^′^ from *L* by removing all vertices not connected to *x*. This makes *L*^′^ a set of common neighbors for *R*^′^. *P*^′^ and *Q*^′^ are then formed by eliminating vertices not connected to *L*^′^. *P*^′^ also loses vertices connected to all of *L*. These are added to *R*. If no vertex in *Q*^′^ is connected to all of *L*^′^, then a maximal biclique (*L*^′^,*R*) has been found. A recursive call is made with 〈*L*^′^,*R*^′^,*P*^′^,*Q*^′^〉. *x* is removed from *P* and added to *Q*. The process stops when either *P* is empty or a vertex in *Q* is connected to all of *L*. An example of the search performed by MBEA is depicted in Figure 4.

#### 

**Theorem 1.** MBEA finds all maximal bicliques in a bipartite graph.

#### 

*Proof*. MBEA explores the entire search space of all the subsets of one vertex set and finds all the bicliques by Observation 1. It checks their maximality by Observation 2. It eliminates only those that cannot lead to other maximal bicliques by Observations 3 and 4. Therefore, upon termination, MBEA has found all maximal bicliques.

### Improvements to MBEA

We seek to improve MBEA in two ways: by an early removal of vertices from the candidate set, and by a selection of candidate set vertices in non-decreasing order of common neighborhood size. Both actions tend to help prune the recursion tree by avoiding the generation of non-maximal nodes.

#### Tree Pruning

Recall Observation 3, which asserts that if *P* contains a subset *S* of vertices that are adjacent to all vertices in *L*, then (*L*,*R* ∪ *S*) is a maximal biclique. Our first modification is based on an extension of this observation. Although it suggests the addition of candidates whose neighborhoods contain that of *x*, upon recursive return MBEA treats vertices in *S* just as it does other vertices in the candidate set. That is, every vertex in *S* is still selected for expansion, even though some will lead to non-maximal subsets only. The generation of such branches can be avoided if we subdivide *S* into two subsets as follows. For any *v* ∈ *S*, either the neighborhood of *v* is a proper superset of the neighborhood of *x* (i.e., *N*_*L*_(*v*) ⊃ *N*_*L*_(*x*)), or its neighborhood is exactly the same as that of *x* (i.e., *N*_*L*_(*v*) = *N*_*L*_(*x*)).

**Figure 5:**
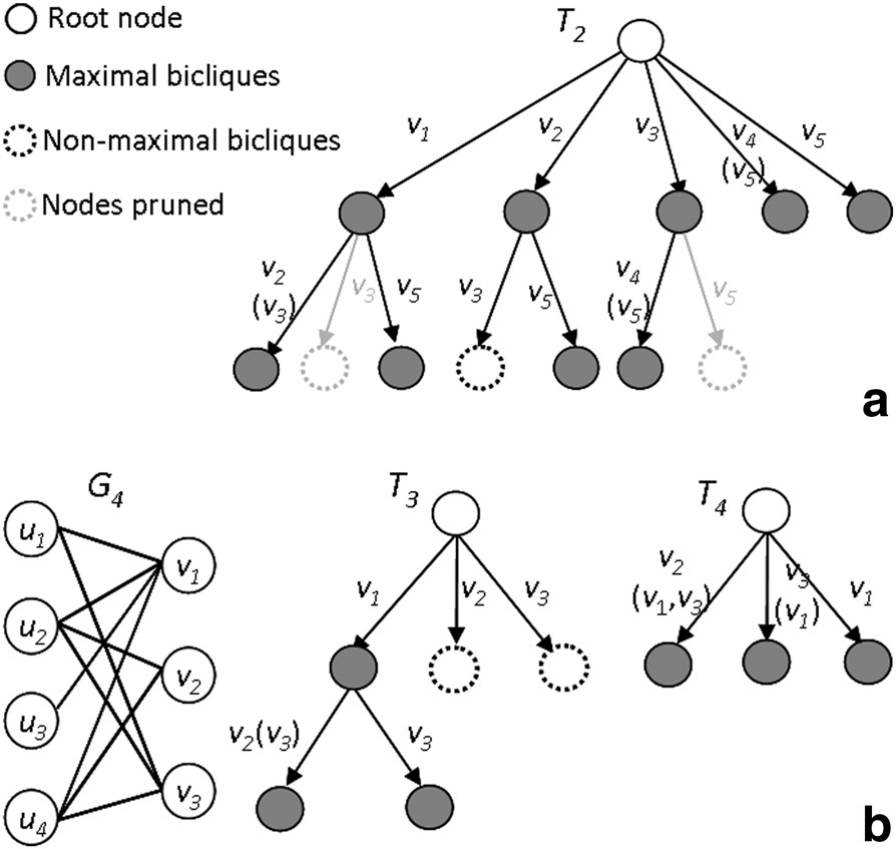
**Improved MBEA (iMBEA).** Examples of MBEA improvements: **(a)** a recursion tree *T*_2_ using Observation 5 on *G*_3_ of Figure 4, and **(b)** the recursion trees *T*_3_ and *T*_4_ without and with the candidate selection method on *G*_4_.

Vertices of the second group can thus be moved directly to *Q* upon recursive return, because any biclique that excludes *x* but includes *v* is a subgraph of a biclique including both *x* and *v*. See Figure 5 for an example. This construction is formulated as follows:

##### 

**Observation 5.** *Any vertex in P with neighborhood L must be an element of the current biclique, and thus can be added to R. Otherwise, any biclique in the current subtree is non-maximal.*

#### Candidate selection

Observe that MBEA chooses candidates in given (arbitrary) order. The second modification we consider was inspired by noticing that leftmost branches, which are explored earlier, generally have more candidates to generate sub-branches than do rightmost branches, which are searched later, as long as the selected candidates have the same number of connections to *L*.

Consider for example a connected bipartite graph *G*_4_ = (*U* ∪ *V*,*E*) where |*U*| = 4,|*V*| = 3 and vertex *v*_1_ ∈ *V* is adjacent to all vertices in *U* (Figure 5(b)). If *v*_1_ is the first selected candidate, then both *v*_2_ and *v*_3_ are candidates at the same level because both connect to at least one vertex in *U*. Both {*v*_2_} and {*v*_3_} are non-maximal, however, since they are subgraphs of bicliques including *v*_1_. On the other hand, if *v*_1_ is the last selected candidate, then there is no vertex left in the candidate set because *v*_2_ and *v*_3_ have been explored earlier. Vertex *v*_1_ is thus directly added to all bicliques according to Observation 3, since *v*_1_ is adjacent to all vertices in *L*. We conclude that selecting candidates in non-decreasing order of common neighborhood size may avoid generating numerous non-maximal subsets. Moreover, it can lead to more balanced recursion trees, which is an important property in load-balanced parallelization.

#### Improved algorithmic details

**Figure 6:**
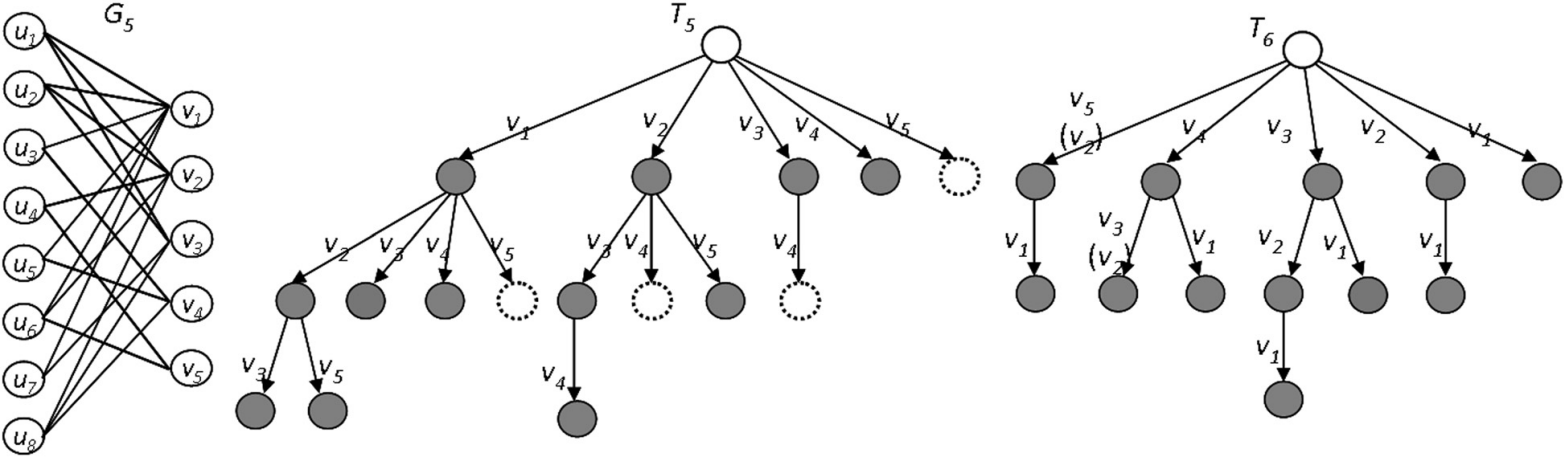
**Recursion trees of MBEA versus iMBEA.** The recursion trees *T*_5_ of MBEA and *T*_6_ of iMBEA for bipartite graph *G*_5_.

To distinguish the basic method from the improved, we shall denote the latter by iMBEA, the version incorporating the two modifications just discussed. In Algorithm: MBEA, these executable additions are indicated with starred lines. The vagaries of data are important, naturally, and so improvements may not always be what they seem. For example, an effective way to create and maintain a candidates list ranked by common neighborhood size is simply to insert a vertex into its proper place in the list. Although well-suited to this particular task, such a use of insertion sort may actually create a tradeoff between the potential time saved in searching versus that spent inserting. To see this, consider that overall time is probably saved in the case of real or synthetic graphs with variable degree distributions. We may actually do better, on the other hand, to turn off this feature on highly contrived instances, especially those such as regular graphs in which all vertices have the same degree. See Figure 6, which shows differences between recursion trees produced by MBEA and iMBEA on a sample bipartite graph.

### Algorithmic complexity

We first consider the time complexity of a brute-force algorithm that examines all subsets of the smaller vertex partition. Let *G* = (*U* ∪ *V*,*E*) denote a bipartite graph, with |*U*| = *m*, |*V*| = *n*, and *m* ≥ *n*. There are 2^*n*^ subsets of *V*. It takes *O*(*mn*) time to find each subset’s common neighbors in *U*. It also takes *O*(*mn*) time to decide maximality. Thus, the worst-case time complexity of this simple scheme is *O*(2^*n*^*mn*).

Similarly, the worst case number of nodes in a recursion tree for MBEA is 2^*n*^, again bounded by the total number of subsets of *V*. At each such node, the time complexity of biclique_find is *O*(*dn*), where *d* is the maximum degree of any vertex in *V* (the maximum number of vertices in *L* is *d*, and the maximum number of vertices in *P* and *Q* is *n*). Thus, the worst case time complexity of MBEA is *O*(2^*n*^*dn*). The total number of subsets examined by MBEA is considerably less than 2^*n*^, however, because branch-and-bound prunes the recursion tree. We shall therefore note that the number of nodes in a recursion tree is at least as large the total number of maximal bicliques, and analyze time complexity in a fashion similar to that performed in [17,27]).

#### 

**Lemma 1.** Every intermediate node in the recursion tree for MBEA represents a distinct maximal biclique.

#### 

*Proof*. Nodes on MBEA’s recursion tree represent maximal or non-maximal bicliques. Without pruning, a non-maximal node may be formed only when a candidate or a former candidate’s neighborhood is a (not necessarily proper) superset of the current set *L*. In the former case, candidate vertices (from *P*) whose neighborhoods contain *L* are automatically added to *R* by Observation 3. Furthermore, if a candidate’s neighborhood exactly equals *L*, then no branching is needed based on Observation 5. The biclique at any intermediate node is thus maximal because further candidate additions would reduce the size of *L* and lead to another maximal biclique. In the latter case, a former candidate whose neighborhood contains *L* leads to no more maximal bicliques from that branch. A non-maximal node with a former candidate connected to all vertices in its *L* is therefore a leaf. We conclude that all intermediate nodes in the recursion tree are maximal.

#### 

**Theorem 2.** *Given a bipartite graph G* = (*U* ∪ *V*,*E*) *where* |*U*| = *m*,|*V*| = *n*,*m* ≥ *n*, *and* |*E*| = *e*, *the time complexity of the Maximal Biclique Enumeration Algorithm for finding all maximal bicliques in G is*O(eB), where *is the number of maximal bicliques. The time complexity per maximal biclique is O*(*e*)*.*

#### 

*Proof*. As proved in Lemma 1, MBEA expands only the nodes that are maximal bicliques on the recursion tree, which means it creates only maximal bicliques as intermediate nodes on the tree and non-maximal bicliques can only be leaf nodes. In other words, the number of non-maximal bicliques created on the tree is at most the total number of the leaf nodes. For any intermediate node on the recursion tree, the number of its children that are leaf nodes representing non-maximal bicliques is less than *n* - 1. In the worst case, the number of intermediate nodes is $B=\overset{\left(d-1\right)}{\underset{i=0}{\sum }}{\left(n-1\right)}^{i}$, and the number of leaves is ${\left(n-1\right)}^{d}=O(B)$, where *d* is the maximum degree of any vertex in *V*. Thus, the total number of nodes on the recursion tree is O(B). We showed that the time complexity of biclique_find() is *O*(*dn*). It can be restated as *O*(*e*), since MBEA must scan all edges for maximality and biclique expansion in the worst case. Combining time complexity *O*(*e*) at each node with the total number of nodes in the recursion tree O(B), we obtain a time complexity of O(eB) for the overall algorithm, and a time complexity per maximal biclique of *O*(*e*).

To understand the algorithmic complexity a little deeper, we view MBEA under the concept of *delay time*, which we define as in [31] as the running time between the output of two consecutive maximal bicliques. In this framework, MBEA is a “polynomial delay time algorithm” because the elapsed time between the output of any two consecutive bicliques is polynomial in *d* and *n*.

#### 

**Theorem 3.** *MBEA is a polynomial delay time algorithm with delay complexity O*(*d*^2^*n*^2^)*.*

#### 

*Proof*. MBEA takes *O*(*dn*) time to explore any single node in its recursion tree. A maximal (intermediate) node can have at most *n* - 1 non-maximal neighbors (leaves). Even in the worst case, MBEA must traverse no more than back to the root of the tree to find the next maximal node. The depth of the tree is at most *d*. From this it follows that the delay complexity is *O*(*d*^2^*n*^2^).

#### 

**Theorem 4.** *Given a bipartite graph G* = (*U* ∪ *V*,*E*) *where* |*U*| = *m*,|*V*| = *n*,*m* ≥ *n*, *the worst-case space complexity of MBEA is O*(*min*(*d*,*n*)*m*)*.*

#### 

*Proof*. MBEA uses two vectors to store the two vertex sets of the biclique in each node of the recursion tree. The space for storing them is *O*(*m* + *n*). When *m* > *n*, the space complexity at each node is *O*(*m*). Since the depth of the tree is at most *d*, the overall space complexity of the recursion tree is *O*(*dm*). Meanwhile, MBEA uses bitmap vectors to store adjacency matrix of the input bipartite graph, which requires *O*(*nm*) space complexity. Therefore, the space complexity in total is *O*(*nm* + *dm*) = *O*((*n* + *d*)*m*) = *O*(*min*(*d*,*n*)*m*).

Thus, in the worst case, MBEA’s space complexity is quadratic in the order of the graph. This should not be surprising. Indeed, such a result is the best that can be achieved by any algorithm that stores its entire input, since the input size is determined by the number of edges.

## Implementations and testing

We implemented MBEA and iMBEA and compared them to existing implementations of what should be the two strongest competitors: MICA [27], currently the fastest graph theoretical algorithm for finding bicliques in general graphs, and LCM-MBC [26], currently among the most advanced data mining algorithms for finding pairs of frequent closed patterns, improving upon LCM [25]. An efficient implementation of MICA is available at http://genome.cs.iastate.edu/supertree/download/biclique/README.html. Efficient codes for LCM can be found at http://fimi.ua.ac.be/src/. Version 2 is reported to be the faster of the two available LCM implementations. The authors of [26] graciously provided us with their implementation of LCM-MBC, which we used in our comparisons. MBEA/iMBEA and MICA accept graphs in a simplified DIMACS edge list format. LCM/LCM-MBC is not DIMACS compatible, however, and required us to convert an edge list into an equivalent adjacency list for the smaller bipartite partition. Graphs come in many formats, of course, so we did not charge any time for this simple conversion.

All implementations were compiled on and timings performed under the Ubuntu 12.04 (Precise Pangolin) x64 operating system on a Dell OptiPlex 9010 Minitower with an Intel Core i7-3770 3.4 GHz processor, 16.0 GB DDR3 non-ECC SDRAM memory at 1600 MHz (4 DIMMs), and a 500 GB 7200 RPM SATA hard drive. Only sequential implementations of MBEA, MICA and LCM-MBC were compared, each making use of a single compute core. MBEA and iMBEA were written in C and compiled with the GNU gcc compiler with O3 optimization turned on. The MICA and LCM-MBC implementations were also complied with O3 turned on. The wallclock running times we report include both I/O and computation, but exclude the time taken to print out the maximal bicliques. They are the average of ten, five or three runs for graphs that can be finished within one minute, one hour or three days, respectively. Runs that exceeded three days were killed and omitted from the averages. We employed standard data reduction techniques to reduce the size of bipartite graphs for all methods tested. For example, during pre-processing, two or more vertices with the same neighborhood are merged into a single vertex; this process is reversed at post-processing.

### Biological graphs

We tested the algorithms on biological graphs derived from functional genomics data. One set of graphs, which was extracted from cerebellum data, was created using a matrix of correlation *p*-values for gene expression to phenotypes across strains of mice in a single population [32]. The matrix consists of 45137 genes represented by microarray measures of transcript abundance and 782 phenotypes to which the transcript abundances are correlated. A bipartite graph is obtained by placing an edge only where the correlation *p*-value is at or below some preset threshold. The density of this graph can be varied by adjusting the threshold. The lower the *p*-value threshold, the lower the graph density. To test a wide variety of densities, we created twenty graphs over a range of thresholds, from 0.01 to 0.20, with a step of 0.01.

The second set of graphs, which represent phenotype-gene associations, was created from a correlation matrix between 33 phenotypes and 17539 genes, calculated over a panel of more than 300 mice. For each threshold, a phenotype-gene edge is present if the correlation is at or above the threshold. We created graphs with a range of thresholds, so that the lowest threshold ran in a small fraction of a second and the largest in tens of minutes.

In both sets, edge density increases across the range of thresholds. from roughly 0.2*%* to about 2.5*%* in the cerebellum graphs, and from roughly 6.6*%* to as high as 37.4*%* in the pheno-gene graphs. Computational demands increase even more rapidly, because the number of maximal bicliques tends to grow exponentially with a linear increase in threshold values.

### Random graphs

In addition to biological graphs, we tested iMBEA and LCM-MBC on random bipartite graphs, using two different random graph models. The first is the classic Erdős-Rényi random graph model. Here, we fixed the number of vertices in each partition at 300 and varied the density from 0.1 to 0.28. The density range was selected so that the lowest would run in well under a second and the highest would require several minutes. We also tested graphs with 400 and 500 vertices, but the results were similar enough to graphs with 300 vertices that we omit their discussion.

For the second random graph model, we modified the Erdős-Rényi model so that we could study graphs with both high and low degree variability. The graph generator takes as input these four parameters: the size *m* of the larger partition, the size *n* of the smaller partition, the average vertex degree *μ* in the smaller partition, and the coefficient of variation *CV* of the degrees in the smaller partition. (Recall that *CV* = *σ*/*μ*, where *σ* is the standard deviation and *μ* is the mean.) These specifications were used to assign vertex degrees to the smaller partition. No edges were produced within a partition, of course. The assigned degrees in the smaller partition were used to place edges, selecting each endpoint in the larger partition with uniform probability. For example, if a vertex in the smaller partition had been assigned degree three, then three neighbors for it were uniformly selected from the larger partition.

We created three sets of random graphs with this graph generator. The first set fixed the number of vertices in one partition at 10,000 and in the other partition at 1000, the edge density at 4.5*%*, and varied the *CV* from 0.3 to 1.2. The purpose of this set was to test the behavior of MBEA versus iMBEA when the *CV* is varied, it being our intuition that iMBEA might be better suited to graphs with higher *CV*. The second and third sets of graphs were created to test iMBEA versus LCM-MBC when the relative partition sizes were varied. In one set, the size of the larger partition is fixed at 10,000 and the size of the smaller partition is varied from 100 to 1000. In the other set, the size of the smaller partition is fixed at 500 and the size of the larger partition is varied from 5000 to 50,000. In both sets we used an edge density of 3.0*%*, which provided a wide spectrum of partition sizes while keeping runtimes within reason.

## Results and discussion

In this section, we compare runtimes of the various algorithms. MICA turns out not to be competitive on any of our graphs. We therefore exclude its timings from our presentation. For instance, iMBEA outperforms MICA by more than three orders of magnitude on even modest-sized biological graphs. On a somewhat larger graph, iMBEA finishes in under an hour while MICA runs for over three days without completion. And on the largest graphs, MICA runs out of memory. Thus, we feel it is manifest that MICA does not belong in the same class as algorithms such as MBEA and iMBEA, which are specifically targeted at bipartite graphs. We first concentrate on MBEA and iMBEA on both biological and random graphs in order to demonstrate the performance gained by iMBEA’s improved pruning. We then move on to compare iMBEA and LCM-MBC on two sets of biological graphs and three sets of random graphs.

### Comparison of MBEA and iMBEA

**Figure 7:**
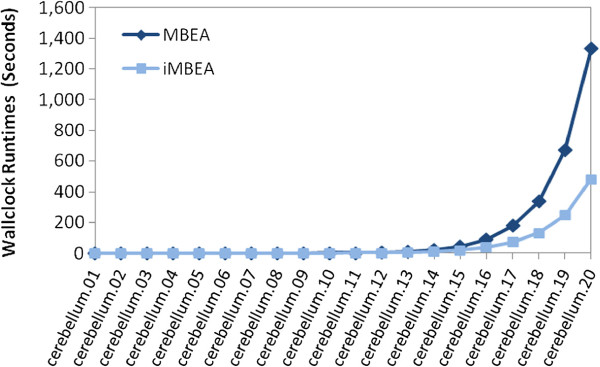
**Performance of MBEA versus iMBEA on biological graphs.** Performance comparison of MBEA and iMBEA on 20 cerebellum graphs from GeneWeaver. As the size and density of the graphs increases, the small overhead incurred by iMBEA’s pruning checks is quickly rewarded with performance gains from the additional pruning.

**Figure 8:**
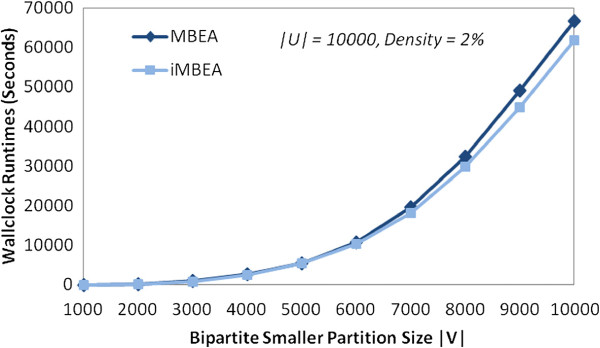
**Performance of MBEA versus iMBEA on random graphs.** Although runtimes are close, iMBEA consistently outperforms MBEA on random graphs.

**Figure 9:**
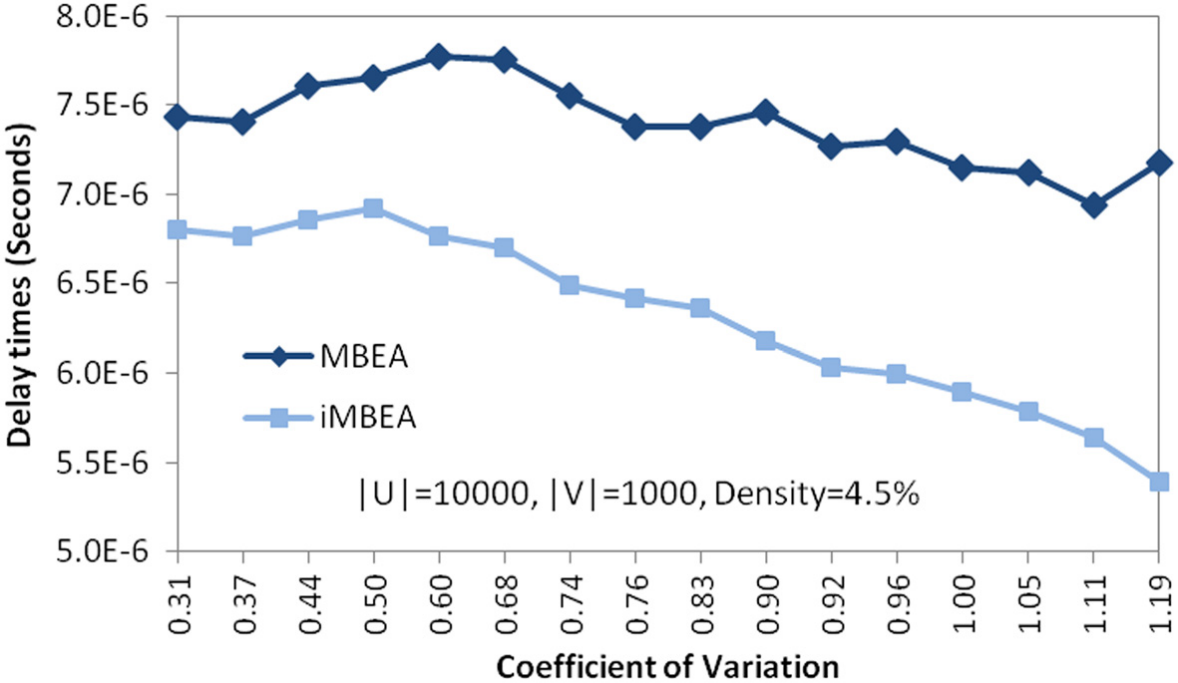
**Effect of graph degree structure on MBEA and iMBEA.** The average delay time of MBEA and iMBEA on random graphs with the same size and density, but varying degree distribution. On graphs with low coefficient of variation, the performance gap between MBEA and iMBEA is narrower than on graphs with high coefficient of variation. This confirms our expectation that the pruning enhancements of iMBEA have a larger effect on graphs with diverse degree structure.

In Figure 7 we compare the runtimes of MBEA and iMBEA on the twenty cerebellum graphs. The curves cross at a *p*-value threshold of about 0.07. iMBEA is roughly three times as fast as MBEA at around threshold 0.20. These results confirm our expectations that the relative simplicity of MBEA wins on sparse graphs produced at lower thresholds, while the improvement overhead of iMBEA more than pays for itself once higher thresholds generate graphs that are sufficiently dense. We also compared MBEA and iMBEA on random bipartite graphs. As shown in Figure 8, while reasonably close, iMBEA consistently outperforms MBEA. The sorted candidate vertex selection and enhanced pruning of iMBEA appear still to produce performance gains. These gains are not as significant, however, as they were for biological graphs. This may be due at least in part to the rather smoothed overall topology of random graphs, as opposed to the uneven density and highly irregular features typically seen in graphs like those in GeneWeaver. To look closer into this behavior, we varied the *CV* with which random graphs were built. We found, as illustrated in Figure 9, that iMBEA outperforms MBEA on random bipartite graphs over the entire *CV* range tested. The performance gap is smaller when the *CV* is low, probably due to MBEA’s relative simplicity and reduced overhead. As the *CV* increases, however, the performance gap between MBEA and iMBEA widens. These results help explain iMBEA’s superior performance on biologically-derived graphs, which very often exhibit high variation in vertex degree. When comparing our algorithms to other methods, we employ only iMBEA for simplicity. It is possible that on some inputs MBEA would do slightly better.

### Comparison of iMBEA and LCM-MBC

**Figure 10:**
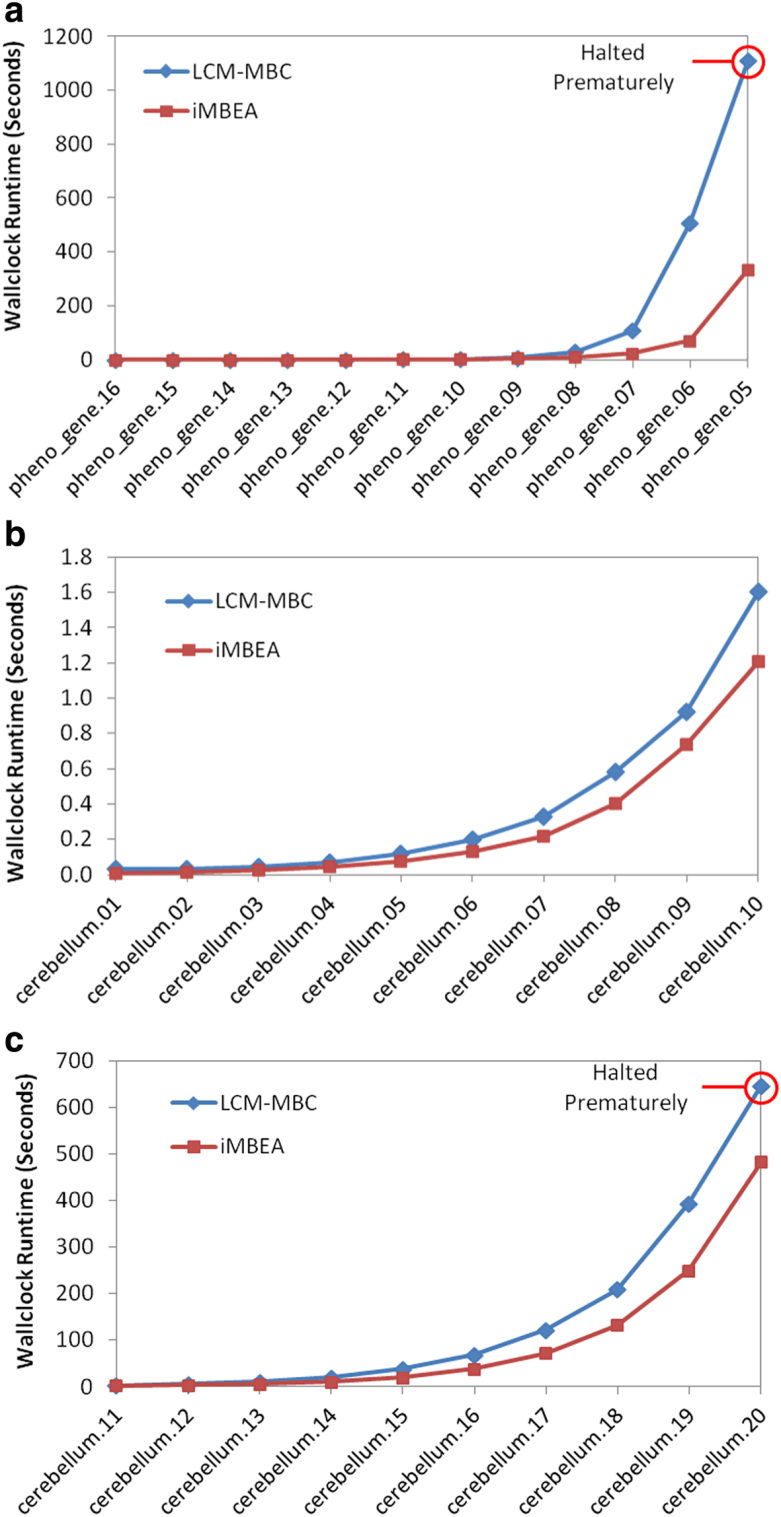
**Performance of iMBEA and LCM-MBC on GeneWeaver graphs.** The GeneWeaver graphs were constructed from two different phenotype-gene similarity matrices. Each edge is either present or absent based on whether it is at or above (or at or below, when *p*-values are used) a given threshold. The graphs in **(a)** were created from a correlation matrix of 33 phenotypes and 17539 genes. Graphs in **(b)** and **(c)** were created from a matrix of correlation *p*-values for gene expression to phenotypes in a single mouse population, using 782 phenotypes and 45137 genes. As the threshold moves to the right along the x-axis, the graphs generally grow larger and denser. The pheno-gene graphs range from 6.6*%* to 34.7*%* density, while the cerebellum graphs range from about 0.2*%* to about 2.5*%* density.

**Figure 11:**
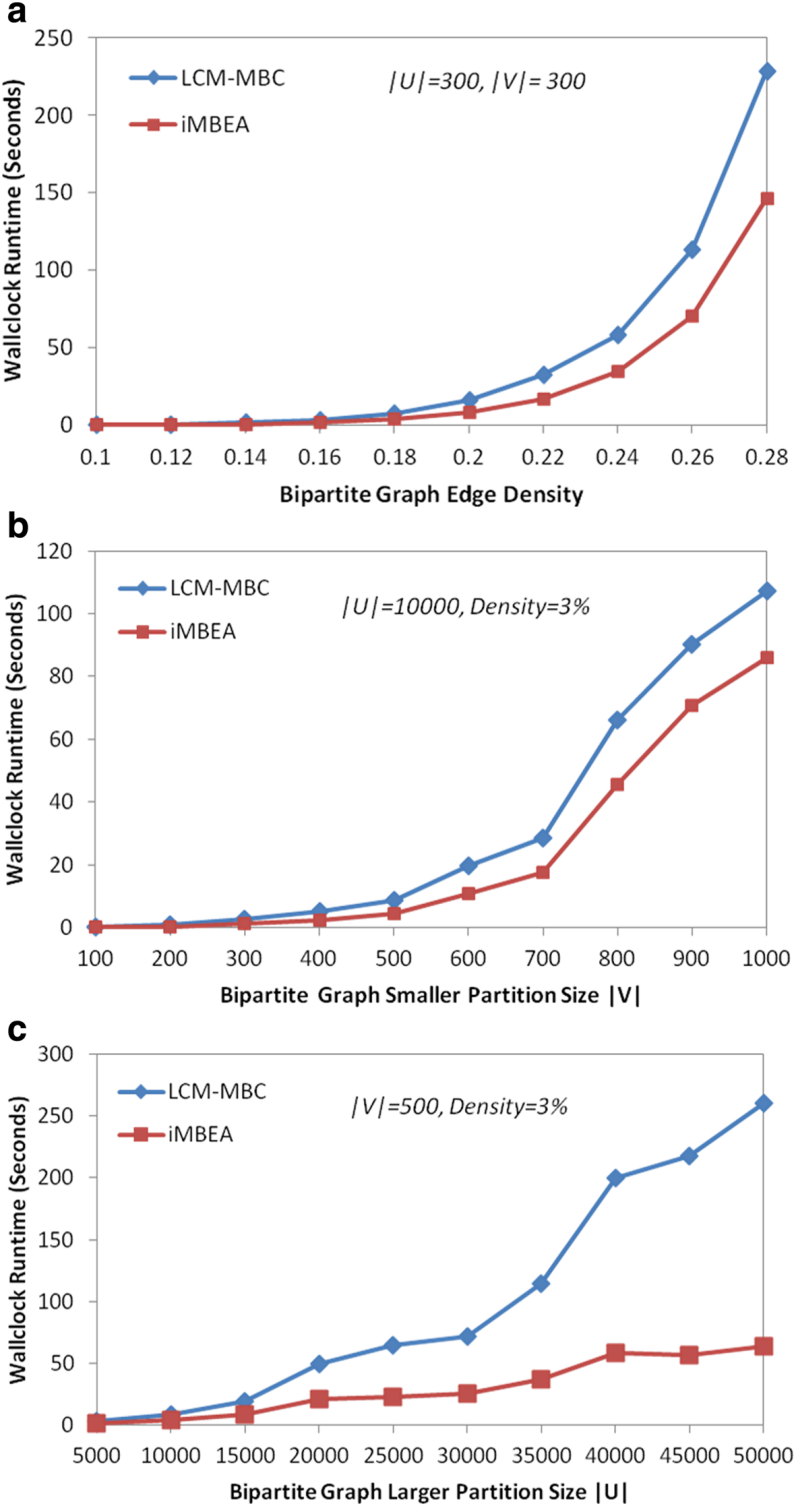
**Performance of iMBEA and LCM-MBC on random bipartite graphs.** The Erdős-Rényi random bipartite graphs in **(a)** have the number of vertices in each partition fixed at 300, but the density is varied from 0.1 to 0.28, showing how density affects runtime. Similar results on graphs with each partition fixed at 400 and 500 vertices are omitted for space considerations. The graphs in **(b)** and **(c)** were generated using the random graph generator described in the text, with *CV* of 1.0 and density of 0.03. In **(b)**, the size of the larger partition is fixed at 10,000 while the size of the smaller partition is varied. In **(c)** the converse occurs; the size of the smaller partition is fixed at 500 while the size of the larger partition is varied. In all three cases, the performance disparity between iMBEA and LCM-MBC is apparent.

Figure 10 shows the average runtimes of iMBEA and LCM-MBC on the biological graphs tested. Part (a) is the pheno-gene graphs, and parts (b) and (c) are two ranges of p-values for the cerebellum graphs. The performance disparity is most notable when the graphs grow denser. On both the cerebellum and pheno-gene graphs, the maximal bicliques in the densest graph exceed the 2 GB disk storage limit of the LCM-MBC implementation, causing the program to halt prematurely, reporting only a portion of the maximal bicliques. The runtime of these two graphs would certainly be much higher if the limit were removed. The results of iMBEA and LCM-MBC on random bipartite graphs are shown in Figure 11. Both methods scale to graphs with thousands of vertices in each partition. The iMBEA algorithm, however, consistently and convincingly outperforms LCM-MBC.

These figures highlight iMBEA’s advantages in scalability. Methods tend not to look very different when graphs are sparse. As data quality improves, however, GeneWeaver and analysis tools of its ilk tend to employ denser graphs in order to capture deeper latent structure. This is where the design enhancements of iMBEA really start to become conspicuous and unmistakable.

## Utility in GeneWeaver

GeneWeaver ( http://geneweaver.org), formerly the Ontological Discovery Environment [11], seeks to identify unique and shared relationships between genes and their roles in biological processes. Aggregated genomic data is integrated, and relevant associations are represented, with discrete bipartite graphs. These allow relationships from diverse experimental sources to be combined. GeneWeaver employs MBEA/iMBEA on these graphs to discover the ontology or structured inheritance of biological processes through the genesets that support them. This is accomplished through an enumeration of maximal bicliques, which are organized as a directed acyclic graph (DAG) to form an empirically derived interpretation of relationships between biological processes. An implementation of this systematic approach, including MBEA/iMBEA, is embedded in the web-based GeneWeaver software platform. Data availability has driven this application to emphasize genes as the primary biological entity through which relationships are inferred. Nevertheless, the model is general enough to map easily onto other biological entities or attributes. Thus, GeneWeaver provides a computationally scalable approach to subset-subset matching in the quest to increase our understanding of molecular networks that support biological function.

### Motivation

A major challenge in bioinformatics is to identify relationships among poorly characterized genes and their varied roles in biological processes, and to group these processes along functionally meaningful lines. For example, one may be interested in whether (and which of) the biological bases of psychiatric disorders such as anxiety or depression are also involved in alcoholism. Each disorder may be attributable to multiple genes, and each gene may be involved in multiple disorders (pleiotropy). Biological processes are typically categorized by ontologists based on their external manifestations. Unfortunately, phenomena such as convergent evolution (when two similar structures or functions are obtained through distinct evolutionary processes) and other factors that result in functional similarity can lead to poor classification schemes that do not map onto the supporting biology. Thus, for basic research into discovery of the biological underpinnings of diverse processes, a classification of biological functions can instead be based on sets of underlying genes. Finite simple graphs are a natural way to represent relationships between such sets. Graph algorithms are a useful tool in their analysis and interpretation. The need to study whole genome versus biological functional data makes bipartite graphs an appropriate model for finding associations between pairs of disparate data types. Enormous correlational structures can arise in data of this size, however, potentially making the task of biclique enumeration a limiting computational bottleneck. This is because classification and assessment of the phenome space is theoretically unbounded, especially in the case of genome-scale ontological discovery. The MBEA and iMBEA methods were therefore developed to harness fast algorithm design techniques and to exploit bipartite graph structure in order to satisfy the staggering computational demands that may be incurred in the creation of emergent phenotypic ontologies.

### Data

A biological pathway or process can be associated with a set of genes. Such a set typically comes from some biological source, for example, an experiment related to drug abuse. Gene sets can be generated with any methodology dedicated to gene-network creation. Commonly used methods include differential gene expression, genetic correlation to gene expression, positional candidates from genetic mapping, associations obtained from text mining, and literature reviews and/or empirical studies in which researchers compile gene lists involved in various behavioral constructs such as pain, aggression, alcoholism and drug abuse.

The GeneWeaver database currently contains over 75,000 gene sets covering nine species: *Caenorhabditis elegans* (roundworm), *Danio rerio* (zebrafish), *Drosophila melanogaster* (fruit fly), *Gallus gallus domesticus* (chicken), *Homo sapiens* (human), *Macaca mulatta* (monkey), *Mus musculus* (mouse), *Rattus norvegicus* (rat) and *Saccharomyces cerevisiae* (yeast). When sets from different species are combined, gene homology is used to match genes onto a set of reference gene ID clusters. Although the gene-set space is unlimited, the genome space is constrained by the finiteness of the genome itself. (The human genome, for example, is currently estimated to contain roughly 20,000-25,000 genes.) It should be noted that the method described here is extensible to include any biomolecule associated with a function or process, including miRNA, transcript forms, gene products and their various states and many additional entities involved in biological processes. Likewise, it is often desirable to use abundance or co-occurrence statistics to relate one class of biomolecules to another, including transcripts and miRNA, or transcripts and proteins. Thus, the size of the biomolecular vertex class is also potentially without bound.

### Model

A biclique-based model was developed to extract functions along with functionally similar genes from gene sets derived from various sources, and then to organize them as a DAG to represent an entire ontology of biological functions. This model consists of three major components: a *combine module* to compute gene-set association matrices to construct bipartite graphs via thresholding and graph mapping, a *biclique module* using MBEA/iMBEA to enumerate maximal bicliques from gene-set bipartite association graphs, and a *phenome graph module* to organize gene sets by integrating maximal bicliques into a DAG to represent an ontology of functions.

### Graphs

The combine module melds gene sets from various sources, computes a real-valued scoring matrix to associate genes with functions, converts the matrix to binary by applying a suitable threshold, and transforms the matrix into a bipartite association graph. Homology may be employed when more than one species is involved. Scoring can be based on a variety of statistical metrics, including correlation coefficients, *p* or *q* values, literature associations and other categorical analyses. Thresholding may be soft or hard, and is generally performed with the aid of low and high pass filters. Keywords such as “drug,” “alcohol” and “cerebellum” are used to select gene sets, based on search term occurrence in metadata. These sets may be fused to form larger collections of putative biological functions.

### Biclique enumeration

The biclique module uses MBEA/iMBEA to enumerate all maximal bicliques in the bipartite gene-set association graph. Here a biclique represents the relationship between a set of biological functions and the genes with which they are commonly associated. Maximality ensures that this relationship is not properly contained within another. A maximal biclique thus denotes a unique set of functionally similar biological processes along with the genes they share in common.

### Ontological integration

The phenome graph module constructs an ontology of functions. Maximal bicliques are connected based on their relationships. The resultant hierarchical similarity graph represents sets of genes associated with common functions. Note that DAGs are similar to hierarchies (forests), except that a child node in a DAG may have more than one parent node. The formulation of the hierarchical similarity graph is based on the following observation, which helps us define an inherent biclique ordering.

#### 

**Observation 6.** *Let P*(*b*) *denote the set of phenotypes in a biclique, b, and let G*(*b*) *denote its set of genes. Given two maximal bicliques b*_1_ and *b*_2_, *P*(*b*_1_) ⊂ *P*(*b*_2_) *if and only if G*(*b*_1_) ⊃ *G*(*b*_2_), *and P*(*b*_1_) ⊃ *P*(*b*_2_) *if and only if G*(*b*_1_) ⊂ *G*(*b*_2_)*.*

We can now define a hierarchical similarity graph using maximal bicliques for nodes and a partial ordering of the bicliques for arcs (directed edges). Node *b*_1_ will be an ancestor of node *b*_2_ iff *P*(*b*_1_) ⊃ *P*(*b*_2_). In this case we say that *b*_2_ is a descendant of *b*_1_. A node with no ancestors is said to be a root. One with no descendants is said to be a leaf. Node *b*_1_ will be a parent of *b*_2_ iff it is an ancestor and there is no other node *b*_3_ for which *P*(*b*_1_) ⊃ *P*(*b*_3_) and *P*(*b*_3_) ⊃ *P*(*b*_2_). In this case we say that *b*_2_ is a child of *b*_1_. Once these relationships have been formed, an arc is placed from a parent to each of its children.

**Figure 12:**
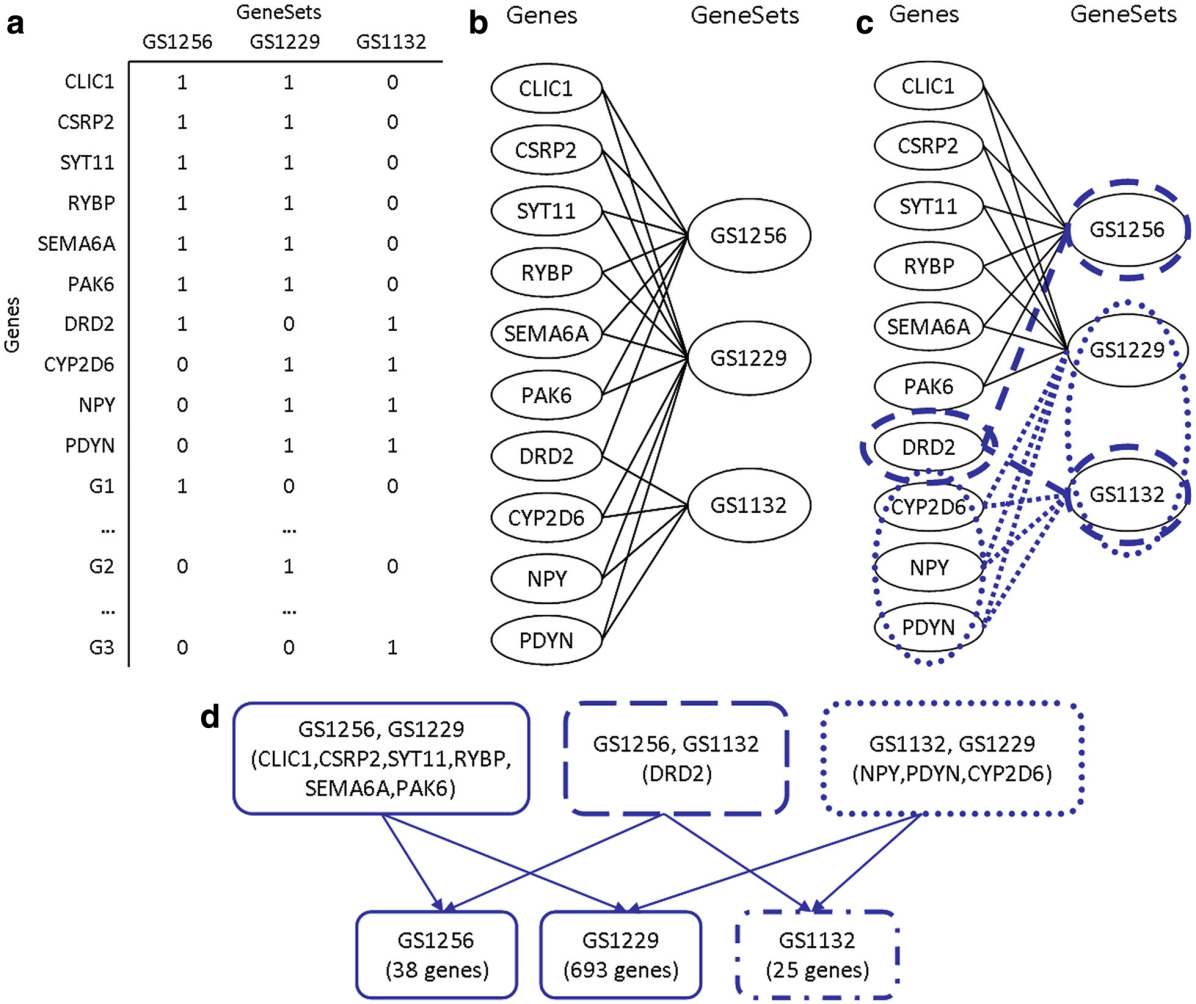
**The hierarchical similarity graph.** Creation of the hierarchical similarity graph of gene sets: **(a)** the source gene sets, **(b)** the gene-set bipartite graph, **(c)** the gene-set bipartite graph with two of its maximal bicliques highlighted, ({*DRD*2},{*GS*1256,*GS*1132}) and ({*NPY*,*PDYN*,*CYP*2*D*6},{*GS*1132,*GS*1229}), and **(d)** integration of all maximal bicliques to a DAG of function relationships. In this graph, the two highlighted maximal bicliques from (b) are roots. The maximal biclique ({25 *genes*},{*GS*1132}) is a child of both, since {*GS*1132} is a subset of the gene sets in both roots. Geneset {*GS*1132} is associated with the genes *DRD*2 and *NPY*, *PDYN*, *CYP*2*D*6 as its parents, but is also connected to 21 genes not shown.

Figure 12 illustrates this construction. A sample hierarchical similarity graph is built from three human gene sets taken from GeneWeaver using the drug-related gene sets listed in Table 2. These sets contain many genes, but we are chiefly interested in the ten genes that are each shared by at least two of the sets. These genes and sets are used to build a gene-set association graph, from which a total of six maximal bicliques are extracted. Despite GeneWeaver’s size and scope, MBEA/iMBEA currently requires at most a few minutes to enumerate maximal bicliques on legitimate queries. A more subtle but equally important task that it must perform is the computation of significance levels for DAG scoring (based on factors such as height and width) among graphs with the same number of genes, gene sets and gene-set associations. Here a re-sampling procedure can be applied to simulate variations in gene-set intersection topology. Such a procedure can easily require tens of thousands of re-sampling operations, however, each needing its own list of maximal bicliques. MBEA/iMBEA can accomplish this task in less than an hour using current technologies, while previous methods were untenable, often consuming several days even on just a few hundred gene sets.

**Table 2:** Gene sets extracted from GeneWeaver

**GeneWeaver IDs**	**Descriptions**	**# Genes**
*GS*1132	Addiction candidate genes derived from literature review [33]	25
*GS*1229	Differential gene expression among heroine and cocaine abusers [34]	693
*GS*1256	Gene expression in hippocampus from human cocaine abusers [35]	38

## Conclusions

We introduced a novel algorithm, MBEA, to enumerate maximal bicliques in a bipartite graph. The technique we described employs efficient branching and pruning strategies to eliminate paths that cannot lead to maximal bicliques. We also presented an improved version of this algorithm, iMBEA, that selects candidate vertices in non-decreasing order of common neighborhood size. Extensive empirical evaluation revealed that iMBEA outperforms MBEA on both biological and random graphs. Furthermore, we tested iMBEA against MICA, a fast consensus algorithm, and against LCM-MBC, a frequent closed itemset data mining method. We observed that both iMBEA and LCM-MBC are orders of magnitude faster than MICA, which we thus eliminated from further consideration. We also found that iMBEA is significantly faster than LCM-MBC, on both random graphs and biologically-based graphs derived from GeneWeaver ( http://geneweaver.org), an online system for the integration of functional genomics experiments. Armed with iMBEA, GeneWeaver provides users with the computational capacity to perform genome-scale analyses of complex relationships derived from diverse biological experiments, with the goal to discover the ontology or structured inheritance of biological processes. MBEA and iMBEA are apt to be well suited to any application in which bipartite graphs can be used to model relationships between two sets of diverse items.

## Availability and requirements

**Project name:** Maximal Biclique Enumeration

**Project home page:**http://web.eecs.utk.edu/~langston/mbea.html

**Operating systems:** Linux

**Programming language:** C

**Other requirements:** None

**License:** GNU-GPL

**Any restrictions to use by non-academics:** No

## Competing interests

The authors declare that they have no competing interests.

## Authors’ contributions

YZ designed and implemented MBEA/iMBEA algorithms and performed timings. CP investigated relationships with frequent itemset mining and performed confirmatory studies. GR aided in algorithm synthesis. EB and EC explored biological applications and oversaw integration into GeneWeaver. ML conceived of the project and directed the analysis. All authors read and approved the final manuscript.
